# Detergent-free purification and reconstitution of functional human serotonin transporter (SERT) using diisobutylene maleic acid (DIBMA) copolymer

**DOI:** 10.1016/j.bbamem.2021.183602

**Published:** 2021-07-01

**Authors:** Marvin V. Dilworth, Heather E. Findlay, Paula J. Booth

**Affiliations:** Department of Chemistry, King's College London, London SE1 1DB, United Kingdom

**Keywords:** SERT, DIBMA, SMA, Transport assay, Membrane protein, Protein thermostability

## Abstract

Structure and function analysis of human membrane proteins in lipid bilayer environments is acutely lacking despite the fundame1ntal cellular importance of these proteins and their dominance of drug targets. An underlying reason is that detailed study usually requires a potentially destabilising detergent purification of the proteins from their host membranes prior to subsequent reconstitution in a membrane mimic; a situation that is exacerbated for human membrane proteins due to the inherent difficulties in overexpressing suitable quantities of the proteins. We advance the promising styrene maleic acid polymer (SMA) extraction approach to introduce a detergent-free method of obtaining stable, functional human membrane transporters in bilayer nanodiscs directly from yeast cells. We purify the human serotonin transporter (hSERT) following overexpression in *Pichia pastoris* using diisobutylene maleic acid (DIBMA) as a superior method to traditional detergents or the more established styrene maleic acid polymer. hSERT plays a pivotal role in neurotransmitter regulation being responsible for the transport of the neurotransmitter 5-hydroxytryptamine (5-HT or serotonin). It is representative of the neurotransmitter sodium symporter (NSS) family, whose importance is underscored by the numerous diseases attributed to their malfunction. We gain insight into hSERT activity through an in vitro transport assay and find that DIBMA extraction improves the thermostability and activity of hSERT over the conventional detergent method.

## Highlights

• The non-aromatic amphipathic polymer DIBMA can be successfully employed to purify human membrane proteins.
• DIBMA solubilisation of hSERT from yeast membranes and resultant nanodisc thermostability is comparable to SMA.
• DIBMA and SMA encapsulated hSERT lipid-nanodiscs exhibit higher binding activity than hSERT DDMCHS micelles.
• Proteoliposomes reconstituted with hSERT-DIBMALPs possess higher transport activity than comparable DDMCHS reconstitutions.

## Graphical abstract

**Figure:**
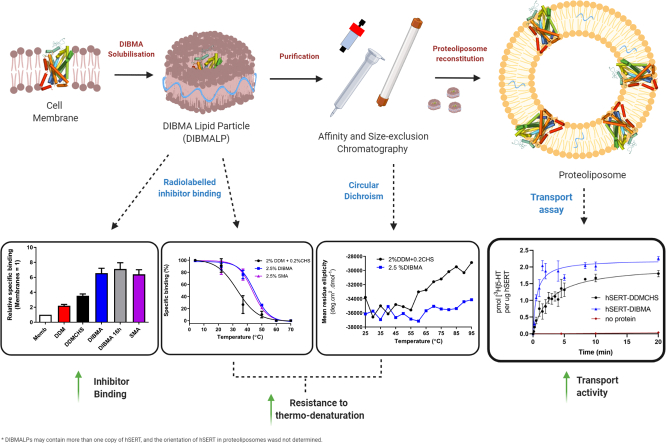

## 1 Introduction

The serotonin transporter (SERT) is a 12-transmembrane-helix integral membrane protein which is a member of the neurotransmitter sodium symporter (NSS) family, along with fellow monoamine transporters such as the dopamine transporter (DAT) and norepinephrine transporter (NET). SERT is responsible for the sodium- and chloride-dependent reuptake of 5-hydroxytryptamine (5-HT, serotonin) into the pre-synaptic cleft throughout the nervous system [1,2]. The serotonergic system is vital and known to modulate numerous cardiological, neuropsychological and behavioural processes. SERT is present in the brain, peripheral nervous system, placenta, epithelium and platelets, and abnormal activity of the transporter has been linked to a myriad of medical disorders including anxiety, depression, autism and obesity [3,4]. Treating these conditions usually involves the use of therapeutic drugs to correctly modulate the transporter [4,5].

Membrane proteins, especially human proteins, are notorious for the bottlenecks associated with their production for structural and/or functional study, and they continue to suffer from problems with overexpression, purification and stability of functional proteins [6]. Current successful recombinant expression systems for the production of hSERT utilise HEK293 cells [7], with the same cell line being used by Coleman et al. in 2016 to finally solve the crystal structure. However, there are difficulties working with mammalian cell expression systems, including problems with amounts attainable and their relatively high expense. Yeast expression provides a cheaper viable alternative that is much easier to scale up. There have been increasing successes using yeast as a recombinant host to alleviate the membrane protein production bottleneck [[8], [9], [10]]. Here, we revisit the possibility of using yeast as a recombinant expression host for hSERT. In particular, we establish a detergent-free method to enable us to stabilise the protein and advance functional investigations through the introduction an in vitro transport assay, which has not previously been achieved for this transport protein.

Expression of recombinant rat SERT (rSERT) in yeast has been previously reported, but rSERT expressed in *P. pastoris* was deemed to be mostly non-functional [11]. However, the conclusion that yeast, specifically the methalotroph *P. pastoris* does not produce functional SERT is premature based on this initial work alone as several reasons could account for the lack of functional rSERT. Firstly, codon usage was not optimised for rSERT in *P. pastoris*. It has been shown that yeast codon bias must be taken into account when designing the expression construct in order to prevent misfolding, incorrect translocation and translational inefficiency among other potential deleterious effects that can negatively affect expression [12]. Codon optimisation was found to be vital for successful expression of mammalian recombinant proteins in yeast to achieve higher and more homogeneous yields [12,13]. The rSERT expressed was also not solubilised from the yeast cell membranes. Earlier studies have shown that mammalian recombinant membrane proteins can often be non-functional when in the yeast membrane and require extraction via surfactants to restore function [14,15]. Maltosides such as dodecyl β-D-maltoside (DDM) have been the most successfully and routinely used for solubilisation of α-helical membrane proteins expressed in yeast [16]. Yeast cell membranes also lack specificity in regard to their native lipid composition, lacking cholesterol and instead having yeast counterpart ergosterol. Therefore it is typically necessary to supplement the detergent with a cholesterol analogue such as cholesterol hemisuccinate (CHS) while extracting recombinant human membrane protein from yeast cell membranes [14,15]. Lack of cholesterol has been shown to significantly impair SERTs ability to transport serotonin and reduce the action of selective serotonin reuptake inhibitor (SSRI) Citalopram [17].

Folding, stability and function of helical proteins is dependent upon the charge, and the chemical and mechanical properties of the lipid bilayer. As a result, extraction of membrane proteins into detergent micelles does not always stabilise functional proteins since detergents cannot replicate bilayer mechanical properties [18,19]. Amphiphilic maleic acid copolymers have emerged as a potentially useful method to extract proteins directly from the membrane, resulting in nanosized lipid particles containing the membrane protein. The most commonly used of these maleic acid copolymers to date, is styrene maleic acid *co*-polymer (SMA) [[20], [21], [22]]. This method of membrane protein extraction is thought to involve the excision of a planar patch or ‘nanodisc’ of lipid bilayer which is subsequently stabilised by the ring of polymer. This process preserves the structure and function of α-helical membrane proteins encapsulated within and results in water soluble, thermostable and monodispersed lipid particles nanodiscs of between 10 and 20 = nm [21,23,24]. SMA has already been employed to successfully purify several human membrane proteins, including the ABC transporters P-glycoprotein, MRP1, MRP4, ABCG2 and CFTR and the G-protein coupled receptor (GPCR), human Adenosine A_2a_ receptor (hA_2a_R). These proteins have been expressed in a variety of different recombinant host cell types including mammalian, insect and yeast [25,26], proving the utility and prominence of SMA as the ‘go-to’ detergent-free method available. However the SMA aromatic styrene moiety limits its use for optical spectroscopy techniques due to a strong UV absorption, hampering protein concentration determination by UV absorbance and far-UV circular dichroism (CD) [24,[27], [28], [29], [30]]. The phenol ring of the styrene moiety is thought to intrude into the lipid bilayer core causing perturbation of the lipid packing order, which might have implications for stability and function of encapsulated proteins [28,29,31]. Additionally SMA is known to precipitate in low concentrations of divalent cations, obfuscating many functional and biochemical protein assays that require Mg^2+^ and Ca^2+^ [24,32,33].

An interesting relatively new SMA-alternative is Diisobutylene/Maleic Acid Copolymer (DIBMA), shown to be an adequate solubiliser of lipid bilayer. This polymer substitutes diisobutylene for styrene and as such has a much lower far-UV absorption. As a result, DIBMA is thought to be a gentle solubiliser with mild impact of lipid acyl-chain order and exhibits resistance to cation-induced precipitation [28,29,34]. DIBMA lipid particles (DIBMALPs) nanodiscs have also been suggested to retain lipid bilayers better than SMA lipid particles (SMALPs), based on a lipid transfer study using fluorescence resonance energy transfer (FRET) [35].

Recently DIBMA has been successfully applied to a number of bacterial membrane proteins expressed in *E. coli*, including the outer membrane phospholipase A (OmpLA) [29], bacterial rhomboid protease GlpG [36], membrane tether protein ZipA and the ATP Binding Cassette (ABC) transporter BmrA [34]. Additionally, the potential use of DIBMA with eukaryotic membrane proteins expressed in yeast and mammalian recombinant hosts has been indicated using crude (unpurified) solubilisations of the G protein-coupled receptors (GPCRs) adenosine A2a receptor (A_2A_R) and calcitonin gene related peptide (CGRP) receptor [34]. However, to our knowledge there is no reported successful purification of a eukaryotic membrane protein using DIBMA, or detergent-free purification and reconstitution of a human transporter for activity studies.

**Fig. 1:**
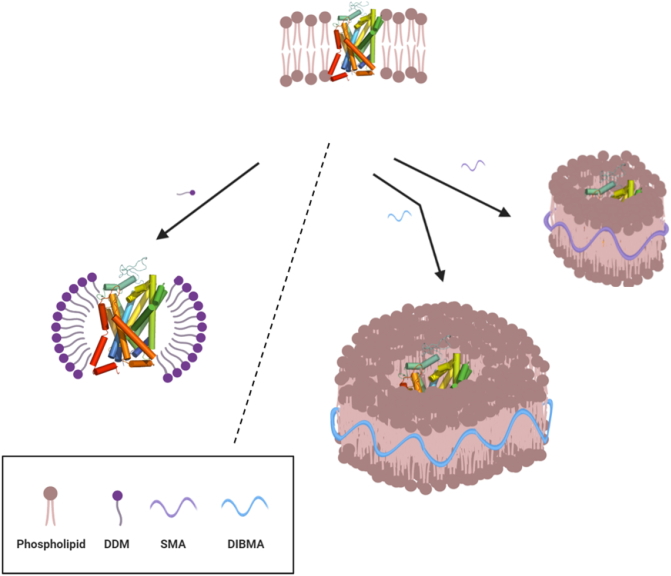
Detergent and detergent-free hSERT membrane extraction. Schematic representation of hSERT extraction from cell membranes using the detergent DDM and the amphipathic maleic acid/copolymers SMA and DIBMA (image created using https://app.biorender.com).

Our goal in this work was to re-investigate the use of yeast as a recombinant host for the expression of hSERT, and to compare the application of the relatively new DIBMA copolymer as an alternative to the well-studied SMA and traditional detergents (Fig. 1). We show that functional hSERT can be obtained with all methods. Since yeast is a cheaper, faster and a more easily scalable approach than insect and mammalian cell lines, we believe that overexpression in yeast and using a detergent-free DIBMA extraction can provide an efficient and convenient method for recombinant eukaryotic membrane protein expression and study. A DIBMA approach also alleviates the instability that can result from detergent solubilisation as well as avoids the often prohibitive costs of employing detergents for solubilisation and purification. Since there is no existing transport assay for purified hSERT, we also provide an in vitro transport assay for detergent and polymer purified recombinant hSERT that is subsequently reconstituted into liposomes.

## 2 Methods

### 2.1 Plasmid construction

A pEX plasmid vector encoding hSERT DNA was synthesised (Eurofins MWG) with the two known N-linked glycosylation sites being abolished by mutagenesis (N208Q and N220Q). The synthesised pEX-hSERT was subcloned into pPICZBα (Invitrogen, discontinued). The resultant pPICZBα-hSERT-His^6^ was modified further through the addition of another four histidine residues, generating the desired 10-his tag final construct pPICZBα-hSERT-His^10^ (referred to as pPICZBα-hSERT).

### 2.2 Transformation, clone selection, expression and culture condition optimisation

Electrocompetent cells of *P. pastoris* strain SMD1163 were transformed with 2 μg *PmeI* linearised pPICZBα-hSERT construct using electroporation. The transformed cells were plated onto YPDS plates with increasing concentrations of zeocin (100, 250, 500 and 1000 μg/mL) and incubated at 30 °C for up to 8 days. Six colonies from each zeocin concentration were screened for expression by culturing in shake flasks containing 50 mL BMGY (Buffered glycerol-complex medium; 1% yeast extract, 2% peptone, 100 mM potassium phosphate pH 6.0, 1.34% YNB (Yeast Nitrogen Base without Amino Acids with Ammonium Sulfate), 0.4 μg/mL Biotin, 1% glycerol) overnight with shaking at 220 rpm and 30 °C. The cells were then transferred to 50 mL shake flasks containing BMMY (BMGY with glycerol replaced with 0.5% methanol) and cultured to an OD600 of 1. Samples were taken after 24 h and membrane preparations and immunoblot analyses were performed, allowing the identification of high-yielding clones. These high yielding clones were scaled up to 500 mL and cultured to 48 h post-induction at 22 °C, 25 °C and 30 °C. Samples of 10 mL were taken at various time-points (0, 16, 24, 40 and 48 h) to allow for measurement of cell optical density (O.D), quantification of total protein yield (by immunoblot densitometry) and inhibitor binding using radio-labelled selective serotonin reuptake inhibitor (SSRI) [^3^H](*R*/*S*)-citalopram.

### 2.3 Membrane preparation

Cell pellets (from 6 × 500 mL cultures grown to around O.D 15–20, ~40-55 g wet cell weight) were resuspended in cold breaking buffer (50 mM Tris-HCL, 150 mM NaCL, 100 mM βME, 0.1 mM PMSF, 5% glycerol, pH 7.4), and one protease inhibitor tablet containing EDTA (cOmplete, Roche) at a 1:1 ratio of cell pellet to breaking buffer. The resuspension was decanted by syringe drop-by-drop into liquid nitrogen, the resultant frozen mini-pellets were broken using a Cryo-mill (6875 Freezer/Mill, SPEX). The resulting cryo-milled dust was thawed on ice and spun at 10,000 × *g* to remove cell debris, with an additional spin at 100,000 × *g* of the supernatant performed to retrieve the total cell membranes fraction. The isolated *P. pastoris* total cell membranes pellet containing recombinant hSERT were resuspended in cold membrane buffer (50 mM Tris-HCl, 150 mM NaCl, 10% glycerol (vol/vol), pH 7.4) to a total protein concentration of 20 mg mL^−1^ determined by bicinchonic acid (BCA) assay. The resuspended membranes were either used immediately or flash-frozen and stored at −80 °C until needed.

### 2.4 Solubilsation

Resuspended *P. pastoris* total cell membranes containing recombinant hSERT at a 20 mg mL^−1^ total protein concentration were solubilised to a final concentration of either; 2% Dodecyl β-D-maltoside (DDM) (w/v) (with or without 0.2% Cholesterol hemisuccinate (CHS) (w/v)), 2% Octyl-β-glucoside (OG) (all purchased from Avanti Polar Lipids), 2.5% DIBMA (w/v) or 2.5% SMA (w/v) and 50 mM Tris-HCl, 150 mM NaCl, 10 mM βME, 0.1 mM PMSF, 10% glycerol (vol/vol), pH 7.4 and one protease inhibitor tablet without EDTA (cOmplete, Roche), made up to a final volume of 20 mL and a total protein concentration of resuspended membranes of 10 mg mL^−1^. The solubilisations were incubated on an orbital rotator, at 4 °C for 3 h for detergent preparations and at either 3 h or 16 h at room temperature for polymer preparations. After incubation preparations were spun at 100,000 × *g* with the supernatant and pellet (resuspended in membrane buffer) flash-frozen or used immediately, allowing further immunoblots for solubilsations efficiency comparisons and purification (with an additional 0.45 μm syringed filter step performed prior to affinity chromatography to remove any remaining contaminants and aggregates that might cause a column blockage). SMA (SMA2000, 2:1 ratio) and DIBMA (Sokolan CP9) were kind gifts from Cray Valley and BASF respectively, and were prepared as described in Lee et al., 2016 and Oluwole et al., 2018 respectively.

### 2.5 Purification

Purification of DDMCHS solubilised hSERT (hSERT-DDMCHS) was carried out by ӒKTA PURE using a 1 mL HisTrap column (GE Healthcare) at 4 °C. The column was equilibrated with 25 column volumes (CV) of buffer A (50 mM Tris-HCl, 150 mM NaCl, 10 mM βME, 0.1 mM PMSF, 10% glycerol (vol/vol), 0.1% DDM, 0.01% CHS, pH 7.4 and 20 mM imidazole). DDMCHS solubilised hSERT containing *P. pastoris* membranes at a concentration of 10 mg mL^‐^^−1^ were loaded on the 1 mL HisTrap which was washed with 25 CV buffer A, and eluted with 15 mL buffer B (50 mM Tris-HCl, 2 mM βME, 0.1 mM PMSF, 10% glycerol (vol/vol), 500 mM imidazole, pH 7.4 supplemented with 0.02% DDM: 0.002% CHS). The peak fractions were analysed by SDS-PAGE and immunoblot, with the desired fractions being pooled, concentrated and further purified by size exclusion chromatography (SEC) or stored at −80 °C until needed.

Purification of DIBMA solubilised hSERT (hSERT-DIBMALPs) was carried out by using gravity-flow column packed with 1 mL bed volume super affinity Ni^2+^-NTA beads (Generon). Beads were first equilibrated with 25 CV buffer A (with DDM and CHS omitted and 5 mM imidazole), the beads were then incubated with 20 mL DIBMALPs solubilised hSERT containing *P. pastoris* membranes at a concentration of 10 mg mL^−1^ overnight on an orbital shaker at 4 °C. The beads were washed with 30 CV Buffer A (with DDM and CHS omitted and 5 mM imidazole), the hSERT-DIBMALPs were then eluted with 5 × 1 mL Buffer B (with DDM and CHS omitted). The eluted fractions were analysed by SDS-PAGE and immunoblot, with the desired fractions being pooled, concentrated, and further purified by size exclusion chromatography (SEC) or stored at −80 °C until needed.

The pooled and concentrated eluates (500 μL) from either 1 mL HisTrap or gravity-flow column purification were further purified by size exclusion chromatography using a Superdex 200 increase 10/300 GL, which was equilibrated with gel-filtration buffer (50 mM Tris-HCl, 2 mM βME, 0.1 mM PMSF, 10% glycerol (vol/vol), pH 7.4) supplemented with either 0.002% DDM and 0.0002% CHS for hSERT-DDMCHS or 0.2 M arginine exclusively for hSERT-DIBMALPs to reduce the non-specific binding.

### 2.6 Radiolabelled inhibitor binding

Saturation binding curve experiments were carried out by adding 200 μg hSERT containing *P. pastoris* membranes to inhibitor [^3^H](*R*/*S*)-citalopram (American Radiolabelled Chemicals, USA) of increasing concentration (0-20 nM), reactions were made up to 200 μL using binding buffer (50 mM Tris-HCl, 150 mM NaCl, pH 7.4) and rotated at room temperature for 3 h (with or without 10 mM unlabelled (*R*/*S*)-citalopram to calculate non-specific binding). At which point the samples were vacuum-filtered using glass fibre filters pre-wet with 0.4% polyethylenimine in binding buffer, captured membranes were washed three times with 5 mL binding buffer and left to dry. The filter-captured membranes were mixed with 3 mL scintillant and counted using a TRI-CARB 1600TR (Packard) scintillation counter, with data being fit to single-site binding curve representing specific binding. The single-point saturation inhibitor binding was carried out by adding 200 μg of total cell membranes containing hSERT or the soluble fraction of the same amount of solubilised membranes to 10 nM [^3^H](*R*/*S*)-citalopram (with non-specific binding calculated as before). With the membrane samples performed using the vacuum filter method described previously, and the solubilised sample having free radioligand removed using Illustra G-50 spin columns (GE). The filter-captured membranes or spin column eluate were mixed with 3 mL scintillant and counted as described previously. All data was processed and fit using GraphPad Prism 8.

### 2.7 Circular dichroism

All CD spectra were measured using an Aviv Circular Dichroism Spectrophotometer, Model 410 (Biomedical Inc.), with specifically adapted sample detection to eliminate scattering artifacts. For full 270–200 nm scans and temperature melts from 25 to 95 °C, circular Suprasil demountable cells (Hellma Analytics) of 0.2 mm path length and rectangular quartz cells of 1.0 mm path length (Hellma Analytics) were used respectively. The protein concentration of 0.4–0.8 mg mL^−1^ was used, and each sample was scanned three times and averaged and the same quartz cells containing only buffer were measured for background subtraction during data analysis. All data was processed using CDTool [37] and GraphPad Prism 8, with the mDeg being converted to mean residue ellipticity (MRE) based upon concentration values derived from UV absorbance at 280 nm for further analysis.

### 2.8 Thermostability assays

For assessment of thermostability via radioligand binding, samples were prepared exactly the same as described for single-point saturation except for an extra preparatory step, the 200 μg hSERT-membrane, hSERT-DDMCHS, hSERT-DIBMALPs and hSERT-SMALPs samples being incubated at a designated temperature in a heat block (4, 22, 37, 50, 60 and 70 °C) for 30 min. Followed by a further 30 min incubation on ice before specific binding was assessed using single-point saturation binding of [^3^H](*R*/*S*)-citalopram (detailed in Section 2.6). For assessment of thermostability via CD a temperature ramp was employed, whereby samples were measured at 222 nm at every 5 °C temperature increment from 25 to 95 °C as the temperature was increased. Loss in 222 nm band intensity was interpreted as loss of α-helical secondary structure. The mean residue ellipticity (MRE) values for hSERT-DDMCHS at 222 nm were used to quantify the loss of secondary α-helical structure as a control, with the MRE at 25 °C being deemed 100% folded protein and at 95 °C representing 0% structure remaining. Allowing comparison to hSERT-DIBMALPs thermostability by comparing α-helical structure loss.

### 2.9 Transport assay

To measure hSERT transport activity, purified hSERT-DDMCHS micelles and hSERT-DIBMALPs were reconstituted into proteoliposomes and their ability to transport substrate [^3^H]5-HT (Hydroxytryptamine Creatinine Sulfate), 5-[1,2-3H(N)]-(American Radiolabelled Chemicals, USA) was measured. The transport assay and proteoliposome preparation was adapted from an earlier reported method used for the SERT homologue and companion neurotransmitter sodium symporter LeuT [38]. Detergent presaturation using OG was used to reconstitute hSERT-DDMCHS [39], and previously documented spontaneous insertion of tetrameric K^+^ channel KcsA-SMALPs into planar lipid-bilayer relied upon to reconstitute hSERT-DIBMALPs into liposomes [27]. Two successful methods have previously been reported for the reconstitution of Cyt*c*O-SMALP nanodiscs into proteoliposomes, relying upon either extrusion or sonication-assisted [40]. We opted for the spontaneous method as opposed to either extrusion or sonication procedure, as this was the most comparable to the detergent counterpart reconstitution and would reduce the variables introduced to allow for a more accurate comparison of subsequent transport activities.

Lipid powder was weighed out and suspended in cyclohexane to achieve a ratio of 20:40:25:15 for CHS, DOPC, DOPE and DOPG (Avanti Polar Lipids) respectively at defined ratio to 10 mg mL^−1^, then heated at 42 °C and freeze-dried overnight. Resultant lipid cake was resuspended in buffer I (50 mM Tris-HCl, pH 7.4) to achieve 10 mg mL^−1^, and stirred for 20 mins. Large unilamellar vesicles were formed by 31 passes through a 100 nm filter in an extruder (Avanti Polar Lipids), and pre-swelled (step omitted for DIBMALPs) with 20% OG to 1% total concentration for 5 min on an orbital rotator. Then purified hSERT-DDMCHS or hSERT-DIBMALPs was added to achieve a 100:1 lipid-protein ratio and incubated for another 1 h rotator, after which point detergent was removed with detergent removal columns (with a mock removal for DIBMALPs) (Thermo Scientific). 20 μL of proteoliposomes were diluted 20-fold, using 380 μL buffer II (50 mM Tris-HCl, 150 mM NaCl, pH 7.4 and 100 nM [3^H^]5-HT) and the reaction incubated for a specified duration (between 0 and 20 min). At which point 5 mL ice cold quenching buffer (50 mM Tris-HCl, 150 mM NaCl, pH 7.4 and 100 μM paroxetine) was added to stop the reaction. The reaction was then filtered under vacuum using glass fibre filters, and washed with 10 mL buffer I. The filters were allowed to dry for 16 h, they were then placed in scintillation vials and 3 mL Ultima Gold MV scintillant (Perkin-Elmer) was added and radioactively measured using a TRI-CARB 1600TR (Packard). A no-protein mock reconstitution was performed in tandem with the protein-containing reconstitutions as a control for the transport assays. This control mock reconstitution consisted of an identical reconstitution procedure but with no purified hSERT present; see Fig. 7. The control sample showed no [^3^H]5-HT uptake indicating that there was no background, passive uptake by the liposome itself.

The concentration of protein in proteoliposomes was determined by a Markwell-Lowry assay [42] assessment of pelleted proteoliposomes. The reconstitution efficiency was calculated as a percentage of protein in the liposome compared to the initial amount of protein added [40,43]. This reconstitution efficiency was factored into subsequent analysis and determination of transport rates and activity, which are thus quoted with respect to the protein concentration in the proteoliposomes. Dot blots (anti-his against hSERT) of reconstituted samples showed that nearly all the hSERT pelleted with liposomes (Supplementary Fig. 2).

CHS was used for liposome preparation instead of cholesterol to maintain consistency with DDM studies, allowing a more direct comparison between the DDMCHS binding and thermostability assays (which required the use of commonly used cholesterol analogue CHS due to it higher solubility) with subsequent transport assays with reconstituted proteoliposomes. We estimated that there was approximately 9 SERT copies per individual liposome (detailed in Supplementary), hSERT orientation in proteoliposomes was not experimentally determined. The initial rates of reaction were defined as being equal to the slope of the tangent line at *t* = 0 for the transport assay curve of pmol [^3^H]5-HT per mg hSERT (*y*) versus time (*t*).

$$lnitial\;rate\;ofreaction=\frac{-dy}{dt}$$

## 3 Results

### 3.1 Optimising expression

**Fig. 2:**
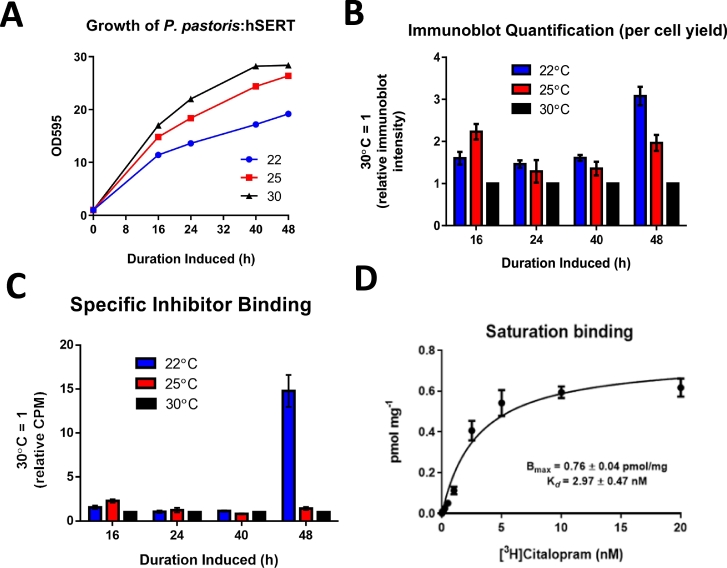
hSERT Expression and optimisation of culture conditions. Post-induction growth curves for *P. pastoris* expressing hSERT cultured at different temperature conditions. Batch seeded 500 mL BMMY *P. pastoris* cultures incubated at either 22 °C, 25 °C or 30 °C post-induction. With 10 mL cultures taken at 16, 24, 40 and 48 h time points to analyse O.D (A), total yield via immunoblot (B) and functional yield via radio-ligand binding assay (C). For the growth data the results were performed three times with similar results with a representative experiment being shown, for the quantification analysis the results are the mean of at least two independent experiments performed in duplicate ± standard error mean (S.E.M). (D) Graph of SERT inhibitor [^3^H](*R*/*S*)-citalopram saturation binding curve to measure functional binding, using 200 μg hSERT containing *P. pastoris* membranes. Allowing hSERT dissociation constant (K_*d*_) (affinity) and maximal binding (B_max_) (functional yield) to be evaluated. Values were derived from non-linear regression specific one-site binding methodology using GraphPad prism, the results represent the mean of three independent experiments performed in triplicate ± S.E.M.

The inhibitor binding of hSERT produced by *P. pastoris* was increased by altering the culture temperature and induction duration, with identical BMMY 500 mL cultures grown at temperatures 22 °C, 25 °C and 30 °C for up to 48 h post-induction. Expression was monitored over the induction period for each temperature condition, with 10 mL samples taken at 16, 24, 40 and 48 h intervals to compare growth rate (tracked by optical density measurements), overall total yield (by immunoblot densitometry) and inhibitor binding (by single-point saturation radioligand binding assay). There was a proportional decrease in growth rate as the induction temperature was decreased from the control 30 °C to 25 °C and 22 °C, with 30 °C being the only culture to that appeared to reach stationary phase pre-48 h induction (Fig. 2A). The total yield expressed for each induction temperature was quantified over a 48 h induction period at 16, 24, 40, and 48 h intervals, by dotblot analysis performed using 25 μg hSERT containing yeast cell membranes extracted from 10 mL samples collected at each interval and quantified by intensity using ImageJ. A significant 3-fold increase was observed at the 48 h interval for 22 °C over the control (Fig. 2B), yet while quantification of total protein yield via immunoblot is useful, there is no guarantee that the improvements in total yield will translate into increased an increase in inhibitor binding. Therefore a complimentary inhibitor binding assay was subsequently undertaken on the membrane preparations at each time interval. A single-point saturation radio-ligand binding assay was performed using radio-labelled SERT inhibitor [^3^H] (*R*/*S*)-citalopram (10 nM). The results showed comparable low binding activity for all induction temperature conditions over the culture period except 48 h at 22 °C, at which point a significant 14-fold increase in binding inhibitor over both the 25 °C and 30 °C conditions was observed (Fig. 2C). Thus a correlative total and inhibitor binding increase was seen at 22 °C for 48 h induction, which was the preferred culture condition used going forward.

### 3.2 Characterisation

#### 3.2.1 Saturation binding of membrane-bound hSERT

A full inhibitor binding saturation curve was performed using 100 μg yeast cell membranes containing hSERT and [^3^H](*R*/*S*)-citalopram with unlabelled (*R*/*S*)-citalopram used to calculate non-specific binding (Fig. 2D), in an effort to assess whether the hSERT expressed was able to bind the inhibitor citalopram and possessed the expected native pharmacological properties. The dissociation constant (*K*_d_) value for (*R*/*S*)-citalopram was 2.97 ± 0.47 nM, which is in the range reported during a similar experiment when expressing hSERT with HEK293 cells (2.1 ± 0.1 nM) [44]. The maximal specific binding or B_max_ of membrane-bound hSERT was evaluated to be 0.76 ± 0.04 pmol mg^−1^.

#### 3.2.2 Solubilisation efficiency, inhibitor binding comparisons and xMA nanodisc size determination

The solubilisation efficiency of detergents DDM and OG (both supplemented with CHS) and maleic acid co-polymers SMA and DIBMA were investigated (Table 1). The insoluble and soluble fractions from each solubilisation were inspected by immunoblot, with ImageJ analysis of the band intensities. The analysis indicated that there was no statistically significant difference in the amount of hSERT extracted from cell membranes, with all solubilising agents releasing between 74 and 81% of total membrane-bound hSERT (DDMCHS 76.1%, SMA 81.3%, and DIBMA 74.2%). This shows that both polymers SMA and DIBMA are comparable solubilising agents to detergents as far as their ability to extract hSERT from the yeast cell membranes is concerned.

**Table 1:** Characterisation and comparison of different solubilisation methods. For each different solubilisation method the amount of hSERT released from the cell membranes, binding to inhibitor [^3^H](*R*/*S*)-citalopram and relative inhibitor binding compared to untreated membranes is described. Solubilisations were performed at 4 °C for detergent preparation and room temperature for polymer preparations for 3 h unless stated otherwise in parenthesis in the condition column. Dynamic light scattering analysis was employed to measure the approximate size of hSERT-xMALP nanodiscs. Results are the mean of at least three independent experiments performed in triplicate ± S.E.M in parenthesis where applicable.

Condition	% Extracted from membrane	Inhibitor binding (pmol mg^−1)^	Relative binding (membranes = 1)
Membrane	–	0.76 (0.04)	1
2% DDM	–	1.7 (0.1)	2.2 (0.2)
2% DDM + 0.2% CHS	76.1 (3.0)	2.7 (0.2)	3.5 (0.2)
2% OG	86.1 (1.5)	–	–
2.5% SMA	81.3 (1.3)	4.9 (0.4)	6.4 (0.6)
2.5% DIBMA	74.2 (6.4)	5.0 (0.4)	6.6 (0.7)
2.5% DIBMA (16 h)	–	5.4 (0.6)	7.1 (0.8)

The maximal specific inhibitor binding of inhibitor [^3^H](*R*/*S*)-citalopram for hSERT in each solubilisation condition was determined (Table 1). A typical 2% DDM + 0.2% CHS (DDMCHS) solubilisation resulted in 3.5-times the binding of isolated yeast cell membranes containing hSERT (membrane-bound hSERT). An approximate 40% reduction in binding was observed when CHS was omitted from the DDMCHS preparation. Solubilisation with 2.5% SMA (RT), 2.5% DIBMA (RT) and 2.5% DIBMA (RT, 16 h) produced a 6.4, 6.6 and 7.1-times increase in binding over membrane-bound hSERT, and approximately 2-fold increase over DDMCHS (Table 1). An ordinary one-way Analysis of Variance (ANOVA) with a tukey's multiple comparisons test was performed using GraphPad Prism 8 software, this determined a statistically significance for all 2.5% xMA binding values over 2.5% DDMCHS (*P* ≤ 0.01) but no significance between individual xMA conditions themselves.

No significant difference in extraction efficiency or binding for hSERT was observed for DIBMA solubilisation at RT for 3 h or 16 h. The yields of purified hSERT-xMALP nanodiscs, especially SMALPs (>0.1 mg/L) were too low for precise Dynamic Light Scattering (DLS) measurements. DLS on supernatant of centrifugally clarified 2.5% xMA solubilisations (100,000 × *g*), gave an approximate average diameter of all extracted disc/membrane fragments of 10 nm and ~19 nm for hSERT-SMALPs and hSERT-DIBMALPs respectively (Supplementary Fig. 3). These diameters compare favourably with those of 10–13 nm for SMALPs and 18–29 nm for DIBMALPs previously reported [29,30, 41].

#### 3.2.3 Purification and CD analysis

**Fig. 3:**
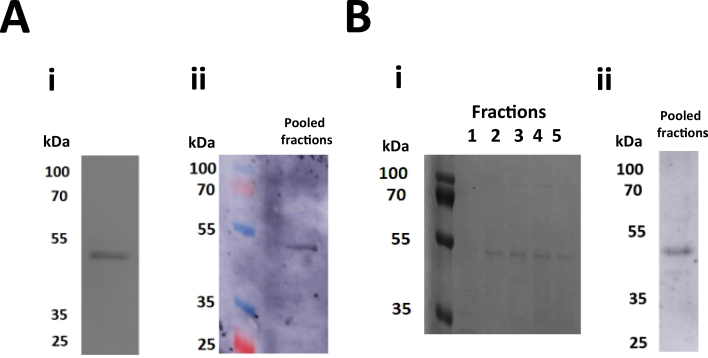
Purification of hSERT by 2-step affinity chromatography. (A) hSERT solubilised with 2% DDM and 0.2% CHS (hSERT-DDMCHS) purified using 1 mL HisTrap and Akta PURE. (i) 12% SDS-PAGE gel stained with coomassie depicted pooled fractions from size-exclusion using Superdex 200 10-300GL confirmed cleaned up hSERT at 50 kDa. (ii) Immunoblot probed with HRP conjugated anti-His antibodies detected a single hSERT band at 50 kDa in the pooled eluted peak fractions. (B) hSERT solubilised with 2.5% DIBMA (hSERT-DIBMALPs) purified using gravity-flow a column packed with 1 mL bed volume Ni^2+^-NTA beads and incubated overnight at 4 °C. (i) Size exclusion chromatography performed using Superdex 200 10-300GL (peak fractions 1–5) were analysed by coomassie stained SDS PAGE, confirming presence of hSERT at 50 kDa. (ii) Immunoblot probed with HRP conjugated anti-His antibodies detected a single hSERT band at 50 kDa in the pooled eluted peak fractions.

**Fig. 4:**
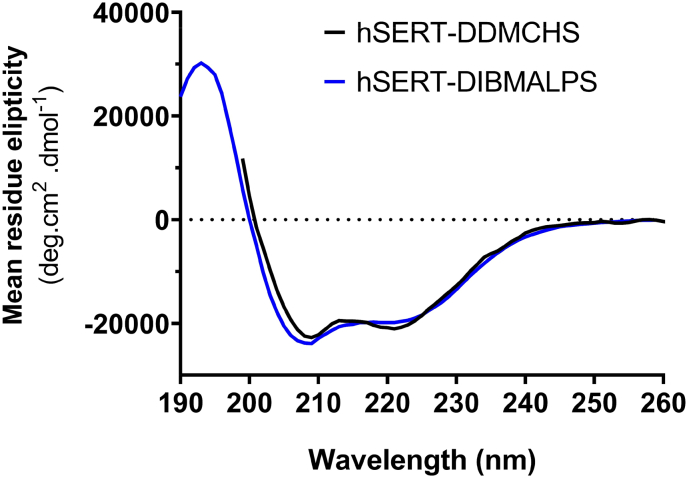
Far-UV circular dichroism (CD) spectra of hSERT. Purified 2% DDM and 0.2% CHS micelles (hSERT-DDMCHS) and 2.5% DIBMA (hSERT-DIBMALPs) was analysed at 270–200 nm by CD spectroscopy. Both spectra have a similar profile with troughs at 208 and 222 nm corresponding with a typically predominant α-helical conformation.

DDMCHS and DIBMA solubilised hSERT was purified using affinity chromatography with the hSERT-DDMCHS micelles purified by 1 mL HisTrap column in conjunction with an Akta PURE and hSERT-DIBMALPs by gravity-flow using a column packed with 1 mL bed volume Ni^2+^-NTA beads (with 16 h incubation at 4 °C). Both were followed by size exclusion chromatography (SEC) using a Superdex 200 10-300GL column (detailed in methods). As shown by coomassie stained SDS-PAGE gel a single homogenous 50 kDa band was detected corresponding to hSERT-DDMCHS in the 500 mM imidazole pooled SEC peak fractions (E) (Fig. 3A.i), this was further verified by immunoblot (Fig. 3A.ii). Gravity-flow column hSERT-DIBMALPs eluate was pooled and concentrated to 500 μL and loaded onto a Superdex 200 10-300GL column (supplemented with 0.2 M Arginine to prevent DIBMA non-specific binding to the column, without which the DIBMALPS would not elute the column reproducibly). Subsequent peak fractions 1–5 were analysed by SDS PAGE confirming the expected 50 kDa band throughout (Fig. 3B.i). The fractions were pooled and verified by immunoblot using anti-His with the same homogenous band present (Fig. 3B.ii). The total purified yield per litre of culture was 0.52 mg/L and 0.21 mg/L for hSERT-DDMCHS and hSERT-DIBMALPs respectively, averaged over 3 preparations (Supplementary Table 2). Representing a 2.5-fold decrease in total yield using the DIBMA methodology rather than the traditional detergent approach. Full wave scan using far-UV CD spectral analysis showed that both detergent-purified and polymer-purified hSERT displayed similar intensities bands at 208 and 222 nm (Fig. 4), indicating a predominantly α-helical conformation as expected of integral membrane proteins.

#### 3.2.4 Thermostability of hSERT-DDMCHS, hSERT-DIBMALPs and hSERT-SMALPs

**Fig. 5:**
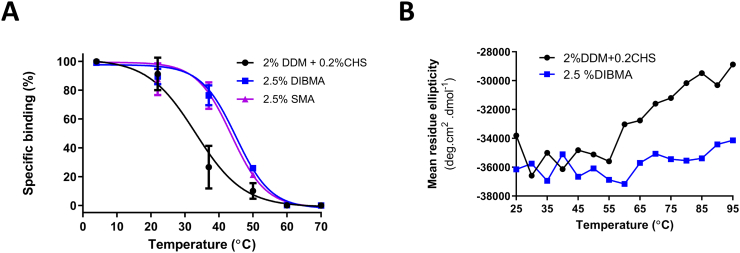
Thermostability of hSERT solubilised in detergents vs. polymers. (A) *P. pastoris* containing hSERT membranes were solubilised with 2%DDM+ 0.2%CHS (black), 2%DIBMA (blue) and 2%SMA (purple), the supernatant was then incubated for 30 min at the indicated temperature then placed on ice for another 30 min before specific binding was assessed by single-point saturation inhibitor binding using 10 nM [^3^H]-citalopram (detailed in 2.6). Results represent the mean of three independent experiments performed in triplicate, relative to the binding at 4 °C ± S.E.M. The T_50_ values are defined as the temperature at which 50% of binding is lost. (B) Thermostability of purified hSERT-DDMCHS micelles and hSERT-DIBMALPs were compared using a circular dichroism temperature ramp (25 to 95 °C) while measuring the loss of 222 nm maxima corresponding to loss of α-helical secondary structure.

To compare hSERT thermostability of xMALPs against DDMCHS micelles we firstly measured the loss in specific binding of inhibitor [^3^H](*R*/*S*)-citalopram after solubilised hSERT had been incubated at a specified temperature (4, 22, 37, 50, 60 and 70 °C) for 30 min, then placed at 4 °C for 30 min prior to the binding assay. *P. pastoris* membranes containing hSERT were solubilised with either 2%DDM + 0.2%CHS, 2.5% DIBMA or 2.5% SMA (all w/v). The hSERT-DIBMALPs and hSERT-SMALPs exhibited superior thermostability over the detergent sample (Fig. 5A), with T_50_ (°C) values of 45 °C and 44 °C respectively, with DDMCHS being only 33 °C. The hSERT-xMALPS retained approximately 70% binding after 30 min at 37 °C, while in contrast hSERT-DDMCHS micelles exhibited a near 3-fold reduction with a remaining binding activity of only 27%.

As membrane proteins are typically predominantly comprised of α-helical secondary structure, we decided to complement the binding assay by measuring the 222 nm band intensity by Far-UV Circular Dichroism, as this particular wavelength corresponds to α-helical secondary structure. The CD signal band intensity of purified hSERT-DDMCHS and hSERT-DIBMALPs were measured at 222 nm to monitor loss of α-helical secondary structure while the temperature on the instrument was increased in 5 °C increments from 25 to 95 °C with a 1 min equilibration period. Upon completion of the temperature ramp a loss in α-helical secondary structure can be seen for hSERT in DDMCHS micelles at 95 °C, whereas hSERT encapsulated DIBMALPs lost only 25% (as determined from the negative 222 nm band intensity at 95 °C) (Fig. 5B).

**Fig. 6:**
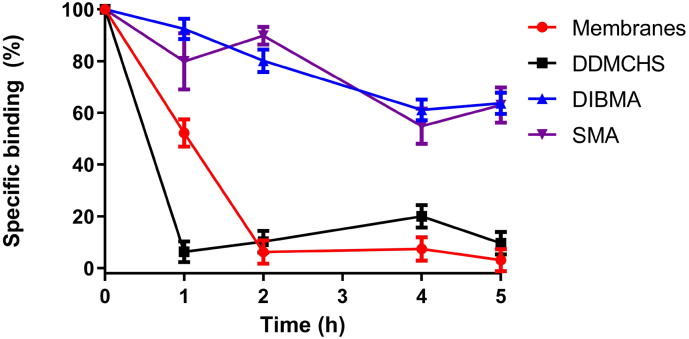
Thermostability comparison of hSERT in different preparations at 37 °C. Either resuspended *P. pastoris* membranes (200 μg) containing hSERT (red) or solubilised with either 2%DDM:0.5%CHS (black), 2%DIBMA (blue) or 2%SMA (purple), incubated for 1–5 h at 37 °C. With the loss of binding assessed by single-point saturation radio-ligand binding using 10 nM [^3^H]-citalopram (described in Methods). Results represent the mean of two independent experiments performed in triplicate ± standard deviation.

The long-term thermostability was measured by comparing [^3^H](*R*/*S*)-citalopram inhibitor binding of membrane-bound hSERT, hSERT-DDMCHS micelles, hSERT-DIBMALPs and hSERT-SMALPs over a 5 h period incubated at the physiologically relevant temperature of 37 °C (Fig. 6). Membrane-bound hSERT and hSERT-DDMCHS micelles retained 49% and 15% binding activity respectively after only 1 h incubation at 37 °C, subsequently losing all binding after 2 h. In contrast hSERT-DIBMALPs and hSERT-SMALPs both retained approximately 90% binding after 1 h, and 60–65% after 5 h, suggesting far superior long-term stability at 37 °C.

#### 3.2.5 5-HT (serotonin) transport

The assessment of recombinant hSERT transport activity produced via heterologous hosts has to date only been determined by inhibitor binding assays using recovered cell membranes, which gives limited information on activity. Additionally, no in vitro transport assay of hSERT has been reported. To gain more insight we developed an in vitro transport assay whereby hSERT transport activity was analysed through measurement of time-dependent [^3^H]5-HT (serotonin) transport across a bilayer. These transport assays involved purifying hSERT with either 2% DDM:0.2%CHS (hSERT-DDMCHS) or 2.5% DIBMA (hSERT-DIBMALPs) and subsequently reconstitution into proteoliposomes. Liposomes with a 20% CHS, 40% PC, 25% PE and 15% PG lipid mix (w/v) and a 100:1 lipid-to-protein ratio were used, together with a no-protein control to assess background signal and liposome structural integrity (described in Section 2.9). The reconstitution efficiencies into liposomes for hSERT-DDMCHS and hSERT-DIBMALPs were similar, being 74% and 79% respectively averaged over 3 reconstitutions (Supplementary Table 1).

**Fig. 7:**
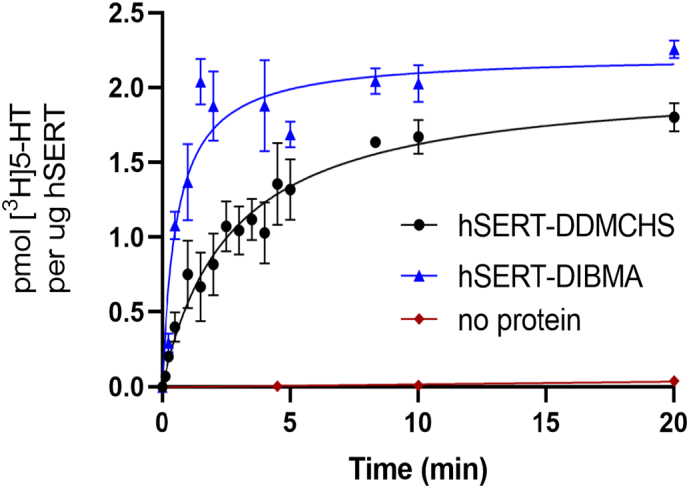
Purified hSERT [^3^H]5-HT(serotonin) transport in reconstituted proteoliposomes. Time-dependent transport of serotonin in hSERT-DDMCHS and hSERT-DIBMA proteoliposomes comprised of 20% CHS, 40% DOPC, 25% DOPE and 15% DOPG with a 100:1 lipid-to-protein ratio. Transport activity was assayed using 100 nM [^3^H]5-HT, with the reactions being halted at each given time-point with cold quenching buffer containing 10 μM Paroxetine. A no-protein mock reconstitution control was performed to assess background signal and liposome structural integrity, all results represent the mean of at least three independent experiments performed in triplicate ± S.E.M with data fit using non-linear regression analysis.

The assay showed that both reconstituted hSERT-DDMCHS and hSERT-DIBMALPs were able to transport serotonin across a lipid bilayer resulting in uptake of serotonin inside the proteoliposome (Fig. 7). Additionally a no-protein control showed negligible transport, supporting the assertion that the only route across the liposome bilayer was facilitation by hSERT. The initial rates of [^3^H]5-HT transport were calculated (as described in Section 2.9) to be 0.7 and 3.5 pmol.mg^−1^. min^−1^ for hSERT-DDMCHS and hSERT-DIBMALPs respectively, representing a 5-fold increase in transport activity when using the DIBMA methodology over traditional detergent. This suggests that some transporter activity maybe lost or is impaired when using detergent to extract hSERT, and that function is preserved in comparison when retaining the local lipid environment and no detergent exposure. The maximal uptakes reported here observed at 20 min incubation of 2.2 and 1.8 pmol μg^−1^ for hSERT-DIBMALP and hSERT-DDMCHS respectively, are comparable to that reported previously for a similar experiment involving the use of vesicles created from isolated human blood platelet membranes (0.9 pmol μg^−1^) [45].

## 4 Discussion

Here, we report the first detergent-free purification and reconstitution of a mammalian transporter, the first purification of a eukaryotic membrane protein using DIBMA and importantly the first in vitro transport assay of purified recombinant hSERT. The transporter extracted using polymer SMA or DIBMA methodology was homogenous, had increased stability and transport activity compared to detergent purification. Reconstituted hSERT-DIBMALPs exhibited superior transport activity over detergent purified protein, which we ascribe to the presence of lipids and the absence of detergent in the polymer methods.

The yeast *P. pastoris* proved to be a useful expression host to obtain functional full length near wild-type hSERT (possessing only two mutations to abolish N-linked glycosylation), with membrane-bound hSERT displaying native-like binding of inhibitor citalopram. By designing our construct with *P. pastoris* codon bias in mind as well as reducing the induction temperature from 30 °C to 22 °C we were able to express homogeneous and functional hSERT. Functionality was verified by saturation inhibitor binding using membrane-bound hSERT, secondary structure via circular dichroism studies on detergent and polymer purified hSERT and serotonin (5-HT) transport assays on the subsequently reconstituted protein.

It was not determined specifically why the 48 time-point and a reduction induction temperature was the ideal conditions for functional hSERT in this study. Early attempts at rSERT overexpression in *P. pastoris* at 30 °C was deemed mostly non-functional [46]. It may be speculated that a slower rate of protein production which a lowered induction temperature (22 °C, as opposed to 30 °C) would cause, allows overexpressed proteins more time to access folding machinery and accessories like chaperones than would be the case using standard conditions. In line with this speculation, we find that the 22 °C culture incubation temperature after methanol induction caused a reduced growth rate over the 48 h induction period, achieving O.D 19 compared to the controls (30 °C) O.D 28 when harvested (Fig. 2A). This resulted in an increased total yield of hSERT as assessed by quantification of hSERT membranes immunoblot bands (anti-HIS) using densitometry (imageJ) (Fig. 2B) and increased inhibitor binding (Fig. 2C) after 48 h when compared to the 30 °C control. A reduced induction temperature is routinely employed to optimise expression of integral membrane proteins in yeast, being thought to reduce the metabolic stress incurred during recombinant expression of exogenous proteins and promote correct folding and insertion into the cell membrane, induce cold-shock chaperones and reduce proteolysis [[47], [48], [49], [50], [51]]. A study on mammalian GPCRs expressed in *P. pastoris*, has similarly reported that lowering the induction temperature to 20 °C (a 10 °C drop from the standard 30 °C) resulted in an increase in inhibitor binding for 10 out of 20 different GPCRs [8]. The hSERT crystal structure shows the presence of a slipknot [52], this is important as similarly knotted proteins have been shown to follow a more complex folding pathway [53]. Thus correct folding of the knot may benefit from a reduced translation rate during induction caused by a drop in culture temperature (from 30 °C to 22 °C). The bacterial homologue of SERT, LeuT, which also contains a slipknot requires a similar reduction in culture temperature for successful over expression [38]. We did not use the chaperone calnexin previously employed with rSERT [54], since there appears to be no benefits for use of calnexin with the human variant (hSERT) [7].

We did not observe significant inhibitor binding until 48 h post induction (Fig. 2C). This lack of inhibitor binding could be attributed to potential high-mannose oligosaccharides species, which have previously been observed to be dominant in the early time points for researchers expressing hSERT using HEK293 cells [7]. Such high-mannose oligosaccharides could explain the poor inhibitor binding as they could result misfolding and non-functional protein that would be degraded. A previous study on hSERT expression in HEK293 cells also found that these unfavourable species decreased by 48 h, with increase in complex glycan species being observed from 32 h onwards. These changes in glycans were ascribed to slow trimming of immature glycans and attaching saccharide components. These potential early time-point unfavourable species may explain the poor inhibitor binding observed in this study prior to the 48 h time-point, due to either nascent proteins failing to fold or being incorrectly glycosylated. As the build-up of non-functioning misfolded proteins in the cell membrane can be potentially damaging to the organism, these unwanted proteins would either be retained by the ER, transported to vacuoles or targeting for degradation [55]. A preliminary colony screening of an enhanced GFP (eGFP) hSERT fusion variant revealed the highest fluorescence and hence highest yield was also seen at the 48 h time-point, this colony also showed no fluorescence at earlier time-points but a significant increase at 48 h post-induction (Supplementary Fig. 4, colony 500.8). As GFP fluorescence is known to correspond to the fusion proteins correct folding and insertion into a membrane [56,57], it could be that hSERT expressed at other time-points may be misfolded. Potentially explaining the poor binding seen in the main study at the earlier time-points, as a result of fewer active binding sites.

Yeast cell membranes lack cholesterol having ergosterol in its place. Typically the production of human integral membrane proteins in yeast requires extraction from the membranes with a detergent supplemented with CHS to recover native-like function [14,15]. Cholesterol depleted HEK293 cell membranes containing hSERT showed reduced inhibitor binding and transport, which reversed upon addition of cholesterol. Such a reversal was not seen with other sterols such as the yeast counterpart ergosterol [17]. Some of the beneficial effects conferred by CHS addition upon membrane protein function are thought to be due to the ordering of lipid acyl tails when packed against the rigid sterol tetracyclic backbone and or/binding to putative cholesterol binding sites [15,58]. A 3.5-fold increase in inhibitor binding was observed for hSERT solubilised in 2% DDM + 0.2% CHS (hSERT-DDMCHS) compared to the membranes-bound hSERT, with omission of cholesterol analogue CHS resulting in a 40% decrease in binding compared to hSERT-DDMCHS (Table 1). hSERT exhibited higher inhibitor binding when solubilised with either polymer DIBMA or SMA (xMA), with approximately twice the binding observed compared to DDMCHS solubilisations, and 6 to 7-fold greater binding than membrane-bound hSERT (Table 1). It appears that the xMA polymers used recover a higher proportion of functional hSERT than their detergent counterparts. The apparent decrease in hSERT inhibitor binding reported in this study, and ability to transport serotonin by other researchers in the absence of CHS in detergent micelles [17], appears to be satisfied when using xMA. Experiments interrogating DDM/CHS micelles using small angle x-ray scattering (SAXS) indicate that DDM-CHS micelles have a shape similar to bicelles, with the DDM and CHS molecules forming a bilayer disc flanked by a belt of DDM molecules [58]. This particular morphology resembles a mini-nanodisc, suggesting this property may be being replicated to an extent in xMALP nanodiscs eliminating the need for CHS. Similar increases in inhibitor binding have also been reported previously for SMA-solubilised human adenosine A_2A_ receptor (hA_2A_R) and β_2_ adrenergic receptor (β_2_AR) expressed using yeast as the recombinant host [16,26].

We observed that DIBMA is as effective as detergent or SMA in isolating hSERT from the yeast membranes specifically, both polymers having comparable solubilisation efficiencies (Table 1). A similar finding has also been reported for bacterial rhomboid proteases extracted from bacterial membranes. Yet a subsequent study saw conflicting data regarding the solubilisation efficiencies for DIBMA ability to extract membrane tether protein ZipA and the ATP Binding Cassette (ABC) transporter BmrA from *E. coli* membranes [34], suggesting the issue might well be protein specific. We also see significantly higher inhibitor binding for xMALPs compared to detergent micelles (Table 1), which we attribute to the fact that hSERT being in a native-like environment throughout the extraction process coupled with the fact that the lipid-nanodisc better mimics the native lipid bilayer of cell membranes. These findings are in line with other reports of similar increases in inhibitor binding seen with SMALPS using the GPCRs human adenosine 2A receptor hA_2A_R [26] and the beta-adrenergic 2A receptor β_2A_R [16] over DDMCHS micelles. We observed increases in inhibitor binding over untreated membranes by both DDMCHS and xMA treated membranes (Table 1). As a result of mechanical cell disruption by cryo-milling, the isolated untreated membranes containing hSERT used for the comparative inhibitor binding studies, are a heterogeneous population of perturbed and less ordered sheared membrane fragments. As this process cracks the cell wall and shears the plasma membrane, organelles such as vacuoles that are known to accumulate overexpressed recombinant protein may remain intact. Potentially leading to reduced access to binding sites for embedded hSERT, when assaying the untreated isolated membranes fraction. Which is not a problem for solubilised membrane protein, as they are typically extracted from the isolated membranes fraction with either detergent or polymer.

The hSERT-xMALPs exhibited superior short-term thermostability when compared to hSERT-DDMCHS micelles with respect to inhibitor binding (Fig. 5A), in line with a similar increased thermostability reported for hA_2A_R-SMALPs [26]. Thermostability analysis using CD to measure secondary structure suggests that although all inhibitor binding was lost after 30 min at 60 °C for all solubilisations, hSERT displayed greater resistance to thermal denaturation with respect to secondary structure while encapsulated in DIBMA than for hSERT-DDMCHS micelles (Fig. 5B). This greater resistance manifested in a 4-fold smaller reduction of α-helical secondary structure in DIBMALPs compared to DDMCHS micelles. Thus the band of polymer band may prevent total thermal unfolding of secondary structure, despite the binding studies indicating that all biologically active binding sites are lost, as suggested by previous researchers [26]. Previously A2aR-DIBMALPs incubated at 4 °C for 6 days reported a 50% reduction in binding, with their SMALPs counterpart retaining 100% [34]. Our mid-term thermostability inhibitor binding assay at the physiologically relevant temperature of 37 °C for 1 to 5 h, indicated hSERT-DIBMALPS and hSERT-SMALPs having near identical stability over the 5 h period and most importantly showed that both hSERT-xMALPs possessed far superior thermostability to DDMCHS micelles. T_50_ value increases of approximately 11–12 °C were seen for both hSERT-xMALPs over hSERT-DDMCHS micelles, in agreement with a similar T_50_ increase of 5 °C reported hA_2A_R-SMALPs [26]. Overall, DIBMA has been proven to be a comparable to SMA in extraction efficiency and stability of hSERT from yeast, and the stability of hSERT at RT and 37 °C while encapsulated in a DIBMALPs provides considerable opportunities for experimentation that are performed at room temperature or biologically relevant temperatures.

Size exclusion chromatography of SERT-DIBMALPs was problematic and resulted in low yields, possibly due to non-specific DIBMA binding to the column (Supplementary Fig. 1C). This was remedied using 0.2 M arginine in the buffer, which has also seen success for other proteins including recombinant monoclonal antibodies (mAbs), human interleukin-6 and basic fibroblast growth factor [59,60]. Both DDMCHS and DIBMA purified hSERT were able to transport serotonin (5-HT) when reconstituted into proteoliposomes. The yield of purified protein per litre was 2.5-fold higher for hSERT-DDMCHS over hSERT-DIBMA (0.52 mg/L as opposed to 0.21 mg/L, Supplementary Table 2), mirroring a similar study which discovered a lower DIBMA purification yield when compared to that of SMA using bacterial membrane proteins ZipA and BmrA [34]. Despite this, hSERT-DIBMALP exhibited a 6-fold higher initial rate of transport than hSERT-DDMCHS. This transport assay highlights the importance of quality over quantity, and the preservation of the local lipid environment and associated lipids for accurate functional studies of eukaryotic transporters. Although it should be noted that the orientation of both the reconstitution hSERT-DDMCHS and hSERT-DIBMALPs was not determined, and may have had an effect on observed transport activities. Due to the difficulty and low yield encountered while purifying hSERT, the potential for implementing mutants for cysteine labelling for the elucidation of orientation was not a viable prospect for this initial study.

DIBMA has significant advantages over SMA, including the tolerance of DIBMA to Mg^2+^ and Ca^2+^ allowing use with a wider range of proteins. DIBMA's branched aliphatic side chain is a gentler solubiliser and unlike SMA does not suffer from lipid bilayer core intrusion by phenyl rings and the subsequent lipid packing order perturbation, or complications that might arise during far-UV and CD techniques. The use of DIBMA can aid protein characterisations, such as far-UV circular dichroism studies. As it will be possible to employ DIBMA extraction of a protein and subsequently reconstitute into proteoliposomes of differing lipid composition, allowing examination of the influence of specific lipids on secondary structure. Additionally, the larger DIBMALP disc is presumable more dynamic due to higher amount of lipid molecules present, which has been posited to better mimic native lipid bilayer than smaller nanodiscs using other polymers for the study of thermodynamics, kinetics and mechanisms [29].

Most studies investigating human transporters typically use mammalian host cell lines for recombinant expression, complemented with whole-cell based uptake assays. The advances detailed here enable the direct in vitro functional comparison of a eukaryotic transporter reconstituted from either detergent micelles or nanodisc lipid-particles, allowing a functional investigation of heterologous recombinant membrane proteins beyond a simple binding assay and where an appropriate cell-based uptake assay might not be viable. The serotonin that is accumulated in the proteoliposomes during in vitro uptake experiments is not in danger of being metabolised or sequestered by intracellular compartments or organelles [45].

## 5 Conclusion

The yeast *P. pastoris* has been shown to be a suitable host for the production of homogenous, folded and functional hSERT, with hSERT exhibiting similar native-like inhibitor binding affinities reported for expression in other cell lines.

Detergent extraction of membrane proteins from the lipid bilayer is imperfect as it strips lipids that may be important for function. Moreover, detergent micelles are thermally unstable and poorly mimic the native membrane environment. Through the employment of the amphipathic maleic acid copolymers SMA and DIBMA, we were not only able to recover a higher proportion of functional protein than via a traditional detergent methodology, but it was also possibly to extract hSERT into a more thermostability and conformationally favourably environment. We find overall that DIBMA and SMA exhibit similar properties for hSERT extraction, including ease of use, functional yield of functional protein and protein stability, but the inherent properties of DIBMA will allow a wider array of biophysical assays to be performed. Furthermore, DIMBA's branched aliphatic side chain is a gentler solubiliser, more generally applicable to a variety of proteins due to its tolerance of divalent cations. The styrene moiety of SMA can present complications due to the phenol rings strong UV absorption and suspected perturbation of lipid packing order due to intrusion into the lipid bilayer core.

We have also developed an in vitro transport assay for hSERT that will enable more detailed studies of the transport mechanism. hSERT displays specific transport activity when reconstituted into proteoliposomes from either hSERT-DDMCHS micelles or hSERT-DBIMALPs. DIBMA solubilisation shows a superior transport activity indicating that detergent-free and lipid retaining extraction, purification and reconstitution is important for preserving function.

To our knowledge, this study details the first purification of a mammalian membrane protein with DIBMA, the first mammalian membrane protein reconstituted using a detergent-free methodology and the first in vitro transport activity using reconstituted purified hSERT.

## CRediT authorship contribution statement

**M**.**V**.**D**: Conceptualization, Methodology, Validation, Formal analysis, Investigation, Writing - Original Draft. **H**.**E**.**F**: Conceptualization, Writing – Review & Editing. **P**.**J**.**B**: Conceptualization, Funding acquisition, Writing - Original Draft.

## Declaration of competing interest

We declare no competing interests.

## Appendix A Supplementary information

Supplementary material Image 1
