# Supplementary material for: Detergent-free purification and reconstitution of functional human serotonin transporter (SERT) using diisobutylene maleic acid (DIBMA) copolymer
Source: Biochim Biophys Acta Biomembr. 2021 Jul 1;1863(7):183602. doi: 10.1016/j.bbamem.2021.183602 (PMC8111416; doi:10.1016/j.bbamem.2021.183602)
Supplement: Supplementary file 1 — Supplementary material [file mmc1.pdf]

SUPPLEMENTARY INFORMATION

Detergent-free purification and reconstitution of functional human serotonin transporter (SERT) using diisobutylene maleic acid (DIBMA) copolymer

Marvin V. Dilworth, Heather E. Findlay and Paula J. Booth

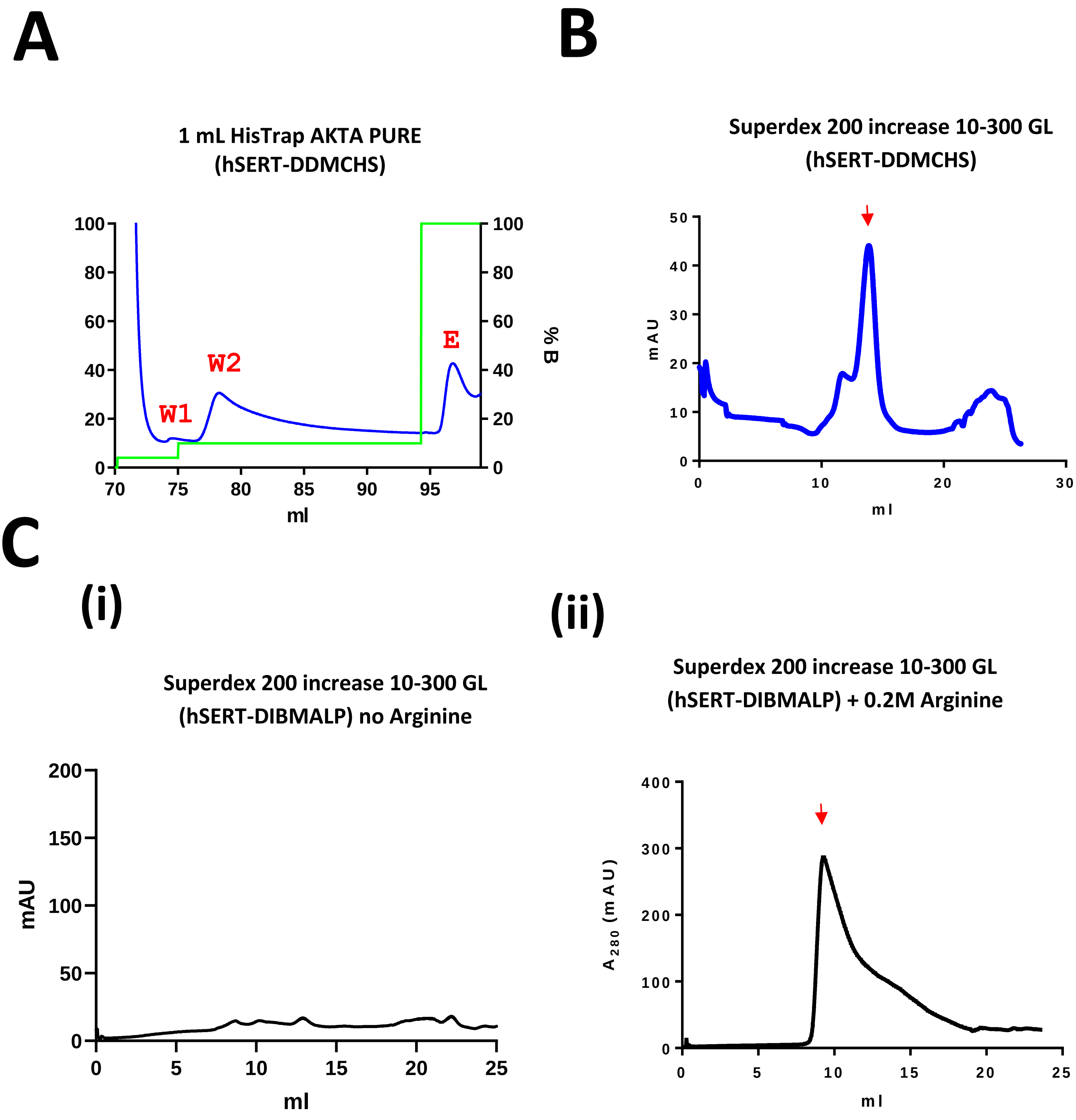

Supplementary Figure. 1. hSERT-DDMCHS and hSERT-DIBMALP Purification. (A) A 1mL HisTrap was used with an Akta PURE using an isocratic elution in the 1st step of the purification of hSERT-DDMCHS (detailed in methods). **(B)** Size exclusion chromatography of the previous hSERT-DDMCHS 1 mL His-Trap elution peak using a Superdex 200 increase 10-300 GL size exclusion column, with the peak fractions (red arrow) concentrated for later coomassie stained SDS-PAGE analysis. **(C)** The pooled eluate from gravity-column purified hSERT-DIBMALPs further purified by Superdex 200 increase 10-300 GL, without (i) and with (ii) the addition of 0.2M arginine to reduce non-specific binding. The peak fractions (red arrow) were concentrated for later coomassie stained SDS-PAGE analysis. Using the GE healthcare multiprotein typical chromatogram for Superdex 200-20 GL, we roughly extrapolated the apparent MW of the of hSERT-DDMCHS and hSERT-DIBMALP appear to be 67 kDa and 669 kDa respectively. This difference in MW may be due to the lipids and polymer in the DIBAMLP sample, and it would be difficult to determine the number of detergent molecules and remaining lipids present for SERT-DDMCHS using an SEC trace also. Additionally, SEC is a hydrodynamic technique that separates molecules in solution not only by size but by shape. Proteins that exhibit elongated shapes thus elute at positions that do not correspond to their dimensions and differ to the position of spherical proteins of the same MW (eg Ca2+ sensor proteins) [1]. Therefore extrapolating the size of a nanodisc using a calibration chromatogram of protein-micelles complexes (in the absence of protein-nanodisc standards) is likely to give inaccurate protein MW. There might also be DIBMA-matrix interactions (as evidenced by the need to add arginine to reduce suspected non-specific binding), which also alter the expected elution profile. As xMALPs purification is in its relative infancy relevant nanodiscs-protein calibration standards and influence of xMALPs on SEC elution is poorly understood.

n	hSERT-DDMCHS	hSERT-DIBMA
1	68	73
2	78	84
3	75	81
avg	74	79

Supplementary Table. 1. Reconstitution efficiencies (%) for hSERT-DDMCHS and hSERT-DIBMA proteoliposomes preparations determined by Markwell-Lowry assay as a percentage of originally added protein. The protein concentration which was successfully reconstituted into proteoliposomes was determined by a Markwell-Lowry assay assessment of pelleted (200,000 *x g*) resuspended proteoliposomes, and the reconstitution efficiency calculated as a percentage of protein in the liposome compared to the initial amount of protein added. These reconstitution efficiencies were then factored in during the radioactivity calculations of [3H]5-HT uptake, allowing accurate comparison of hSERT-DDMCHS and hSERT-DIBMA transports per µg of apparent successfully reconstituted purified protein. As both hSERT-DDMCHS and hSERT-DIBMALPs show comparable ~90% inhibitor binding activity at RT (22°C) we expect minimal non-functional aggregates to be spun down with the pellet and thus we assume all protein in the pellet is successfully inserted in liposomes.

n	hSERT-DDMCHS	hSERT-DIBMA
1	0.51	0.19
2	0.40	0.28
3	0.65	0.15
avg	0.52	0.21

Supplementary Table. 2. Quantification of total purified protein recovered from DIBMA and DDM-CHS solubilsations. The total amount of purified protein was calculated using a Cary 100 bio UV-VIS spectrophotometer and the application of the Beer-Lambert Law. The determined absorbance of purified hSERT sample at 280 nm, the known path length, extinction coefficient, molecular weight and sample volume allowed for calculation of total purified protein in mg/L for each individual prep.

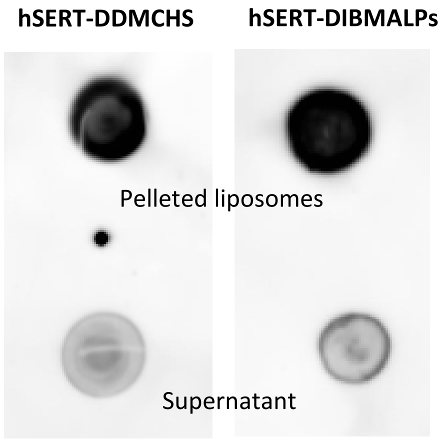

Supplementary Figure. 2. Confirmation of hSERT in liposomes. Proteoliposomes were pelleted at 200,000 *x g*, then subsequently resuspended in buffer A for analysis by dotblot alongside the supernatant using HRP conjugated anti-His antibodies. The vast majority of hSERT was observed in the spun down proteoliposome fraction, confirming the Markwell-Lowry assays.

Calculation of hSERT copies per reconstituted liposome. The concentration of liposomes in the sample was calculated by dividing the molar concentration of lipids by the number of lipid molecules per liposome (approximated from the surface area of a sphere of 100 nm diameter, divided by the average headgroup surface area of the lipids, multiplied by 2 to account for the bilayer). Dividing the final reconstituted protein concentration by this liposome concentration value gives an average number of protein molecules per liposome. This calculation makes various assumptions such as liposome size and distribution in the buffers used. Using this method, we estimated that there is approximately 9 SERT copies per individual liposome. hSERT orientation in proteoliposomes, was not experimentally determined.

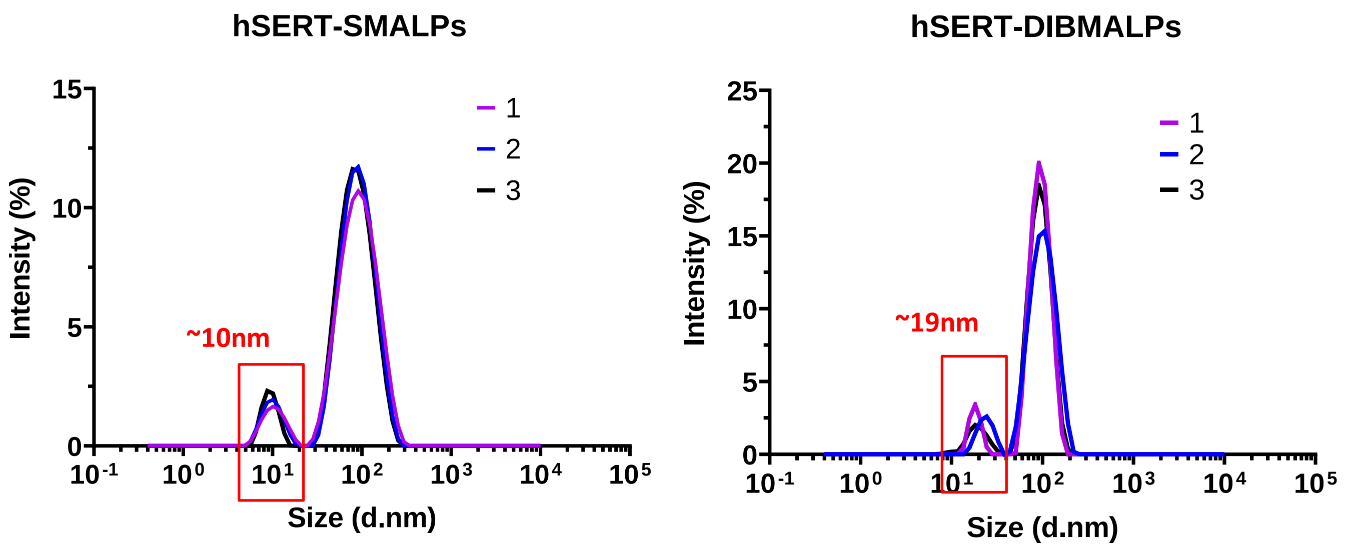

Supplementary Figure. 3. hSERT-xMALP size determination by Dynamic Light Scattering (DLS). DLS measurements were made using a Zetasizer Nano ZS (Malvern). Isolated membranes from P. pastoris containing hSERT were solubilised at 10 mg mL-1 with either 2.5% (w/v) SMA or DIBMA in solubilisation buffer up to a volume of 1 mL. The samples were spun at 100,000 x g and the supernatant used for the measurements. The diameters of the hSERT-xMALPs were then measured in a 1.0 cm path length disposable cuvette (Fisher) at 25°C with 1 min equilibration time. A total of 3 measurements were taken using independent samples, with each measurement consisting of 6 readings which were averaged. The yields of purified hSERT-xMALP nanodiscs, especially SMALPs (>0.1mg/L) were too low for precise DLS measurements. DLS was thus performed on centrifugally clarified solubilsations without further purification (red rectangles), to estimate the average diameter of nanosized disc population. The nanodisc average sizes although from a mixtures of membrane fragments were consistent with the literature, being ~10nm and ~19nm for hSERT-SMALPs and hSERT-DIBMALPs respectively.

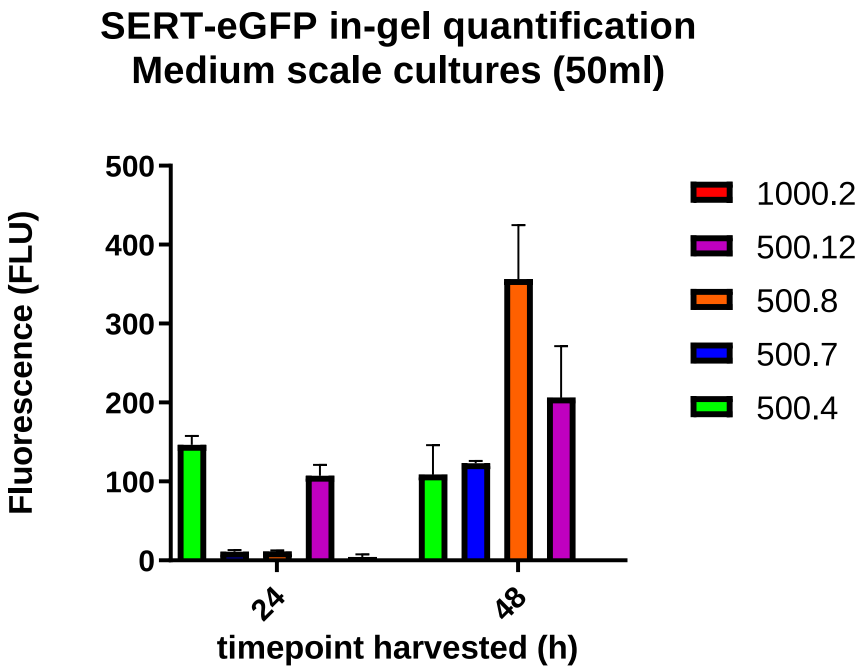

Supplementary Figure. 4. Colony screening of *P. pastoris* SMD1163 hSERT-eGFP transformed cells to determine expression yield assessed using fluorescence. 50ml BMMY cultures were inoculated to an OD 1 and cultured for either 24 or 48 h. At which point cells were harvested, membranes isolated and fluorescence measured using 25 ug total protein concentration of membranes (determined by BCA assay). The highest yielding clone being 500.8, determined by fluorescence, as GFP fluorescence signal is known to correlate and be dependent upon correct folding an insertion into the cell membrane of the fusion partner. 500.8 showed no fluorescence at earlier time-points, yet a significant increase is seen at 48h. This mirrors the specific inhibitor results seen in figure. 2.C. Results are the mean of two experiments performed in duplicate ± S.D.

References

[1] V. La Verde, P. Dominici, A. Astegno, Determination of Hydrodynamic Radius of Proteins by Size Exclusion Chromatography, Bio-Protocol. 7 (2017) e2230. https://doi.org/10.21769/BioProtoc.2230.
